# Supplementary material for: Edges are all you need: Potential of medical time series analysis on complete blood count data with graph neural networks
Source: PLoS One. 2025 Jul 8;20(7):e0327636. doi: 10.1371/journal.pone.0327636 (PMC12237013; doi:10.1371/journal.pone.0327636)
Supplement: S1 Note — (DOCX) [file pone.0327636.s001.docx]

**S1 Note | Potential relevance of complete blood count data to sepsis**

**White blood cells (WBC).** WBC are indicators for infection and inflammation. They include several cell types including lymphocytes, monocytes, neutrophil, eosinophile and basophile granulocytes [1]. During sepsis, neutrophils go through different functional alterations (e.g., altered antimicrobial activity) which can lead to worsening of sepsis [1,2] [REF]

**Platelets (PLT).** PLT have several functions including their contribution in homeostasis and their contributions to the innate immune response to infection and inflammation [3]. Low platelet counts (thrombocytopenia) were shown to be correlated with adverse outcomes in sepsis [1,4].

**Hemoglobin (HGB).** Hemoglobin is responsible for oxygen delivery to the tissue. Patients with sepsis commonly have decreased hemoglobin levels (anemia) [1,5].

**Red blood cells (RBC).** RBC are also responsible for the oxygen transport from lungs to tissue. Additionally, they have important functions in cellular blood immunity. Commonly, septic patients have a decreased RBC count [1,6] [REF].

**Mean corpuscular volume (MCV).** MCV measures the average size and volume of circulating RBC and help in the classification of anemia (microcytic, normocytic or macrocytic). Sepsis is often associated with normocytic anemia [1].

**References**

**1**. Agnello L, Giglio RV, Bivona G, Scazzone C, Gambino CM, Iacona A, et al. The Value of a Complete Blood Count (CBC) for Sepsis Diagnosis and Prognosis. Diagnostics (Basel). 2021; 11. Epub 2021/10/12. doi: 10.3390/diagnostics11101881 PMID: 34679578.

**2**. Resende CB, Borges I, Gonçalves WA, Carneiro R, Rezende BM, Pinho V, et al. Neutrophil activity in sepsis: a systematic review. Braz J Med Biol Res. 2020; 53:e7851. Epub 2020/10/21. doi: 10.1590/1414-431X20207851 PMID: 33111742.

**3**. McDonald B, Dunbar M. Platelets and Intravascular Immunity: Guardians of the Vascular Space During Bloodstream Infections and Sepsis. Front Immunol. 2019; 10:2400. Epub 2019/10/11. doi: 10.3389/fimmu.2019.02400 PMID: 31681291.

**4**. Claushuis TAM, van Vught LA, Scicluna BP, Wiewel MA, Klein Klouwenberg PMC, Hoogendijk AJ, et al. Thrombocytopenia is associated with a dysregulated host response in critically ill sepsis patients. Blood. 2016; 127:3062–72. Epub 2016/03/08. doi: 10.1182/blood-2015-11-680744 PMID: 26956172.

**5**. Docherty AB, Turgeon AF, Walsh TS. Best practice in critical care: anaemia in acute and critical illness. Transfus Med. 2018; 28:181–9. Epub 2018/01/25. doi: 10.1111/tme.12505 PMID: 29369437.

**6**. Effenberger-Neidnicht K, Hartmann M. Mechanisms of Hemolysis During Sepsis. Inflammation. 2018; 41:1569–81. doi: 10.1007/s10753-018-0810-y PMID: 29956069.
