# Supplementary material for: Edges are all you need: Potential of medical time series analysis on complete blood count data with graph neural networks
Source: PLoS One. 2025 Jul 8;20(7):e0327636. doi: 10.1371/journal.pone.0327636 (PMC12237013; doi:10.1371/journal.pone.0327636)
Supplement: S2 Fig — (DOCX) [file pone.0327636.s002.docx]

| 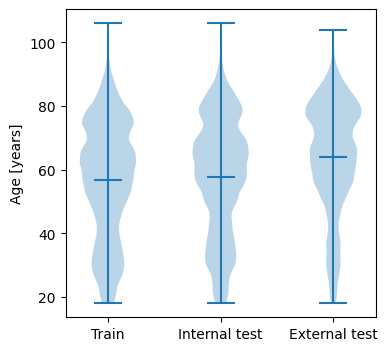 | 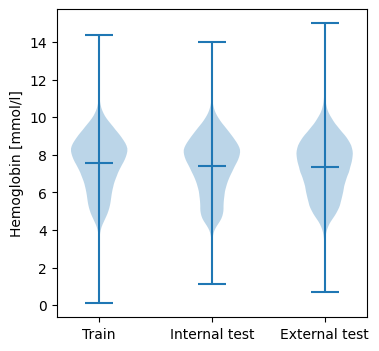 |
| --- | --- |
| 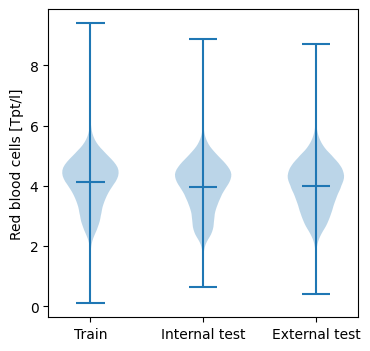 | 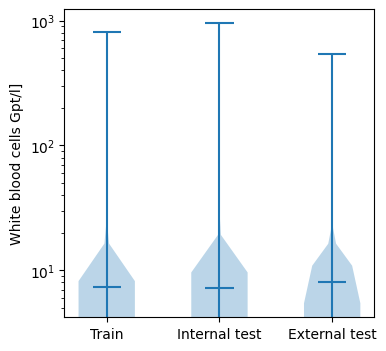 |
| 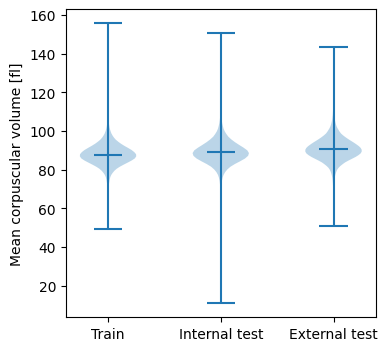 | 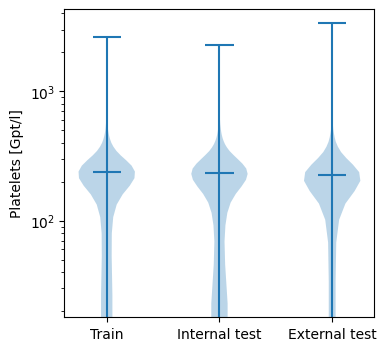 |

**S2 Fig | Distribution of each continuous feature in the train, internal and external test datasets as violin plots.**

We have plotted the violin plots (with minimum, mean, and maximum value) of the feature age (in years), hemoglobin (in millimole per liter), red blood cells (in terra-particles per liter), white blood cells (in giga-particles per liter), mean corpuscular volume (in femto-liter) and platelets (in giga-particles per liter) for the used train, internal test and external test dataset. White blood cells and platelets were semilogarithmic plotted for a better visualization of their distribution. The respective minimum values of white blood cells and platelets are in each case 0 Gpt/l. Note, that we have not visualized the feature “sex” since it is a categorical feature (i.e., either “male” and “female” were reported).
