# Supplementary material for: Edges are all you need: Potential of medical time series analysis on complete blood count data with graph neural networks
Source: PLoS One. 2025 Jul 8;20(7):e0327636. doi: 10.1371/journal.pone.0327636 (PMC12237013; doi:10.1371/journal.pone.0327636)
Supplement: S3 Table — (DOCX) [file pone.0327636.s003.docx]

**S3 Table | Evaluating the robustness of GNNs compared to benchmarks by adding 10 or 100 noisy features to the complete blood count datasets for sepsis classification (higher values represent better performance) and the required training time. We evaluated the classification performance on two datasets (internal and external dataset). Bold values represent the best values in each row. Note that the information of the original dataset (Table 1) was included in this table for a better comparison against the noisy datasets.**

| Dataset | Metric | Set | Tree-based Benchmarks | | | | Non-tree-based algorithms | | Graph learning algorithms | |
| --- | --- | --- | --- | --- | --- | --- | --- | --- | --- | --- |
|  |  |  | **Decision Tree** | **Random Forest** | **RUSBoost** | **XGBoost** | **Logistic Regression** | **Neural network** | **Homogenous Similarity GNN** | **Heterogeneous Similarity GNN** |
| Original dataset | AUROC | Internal | 0.8391 | 0.8700 | 0.8680 | 0.8642 | 0.8369 | **0.8806** | 0.8741 | 0.8747 |
|  |  | External | 0.7870 | **0.8178** | 0.8153 | 0.8121 | 0.7558 | 0.8145 | 0.8052 | 0.8176 |
|  | F1- Macro | Internal | 0.4313 | **0.4770** | 0.4701 | 0.4373 | 0.4412 | 0.4479 | 0.4411 | 0.4422 |
|  |  | External | 0.4018 | **0.4609** | 0.4497 | 0.4184 | 0.3736 | 0.4502 | 0.3964 | 0.4020 |
|  | MCC | Internal | 0.0432 | **0.0605** | 0.0576 | 0.0495 | 0.0442 | 0.0521 | 0.0499 | 0.0506 |
|  |  | External | 0.0291 | **0.0385** | 0.0361 | 0.0327 | 0.0222 | 0.0383 | 0.0308 | 0.0326 |
|  | Training Time [s] | Training | 2.00 | 17.36 | 212.88 | **0.54** | 5.97 | 19.97 | 394.69 | 981.10 |
| With 10 noisy features | AUROC | Internal | 0.8378 | **0.8643** | 0.8605 | 0.8619 | 0.8350 | 0.8542 | 0.8617 | 0.8614 |
|  |  | External | 0.7714 | 0.8155 | **0.8178** | 0.8045 | 0.7553 | 0.7917 | 0.8014 | 0.7923 |
|  | F1- Macro | Internal | 0.4297 | 0.4826 | 0.4723 | **0.5152** | 0.4413 | 0.4417 | 0.4463 | 0.4461 |
|  |  | External | 0.4056 | 0.4661 | 0.4537 | **0.4973** | 0.3743 | 0.4449 | 0.4504 | 0.4031 |
|  | MCC | Internal | 0.0435 | 0.0636 | 0.0570 | **0.0733** | 0.0448 | 0.0484 | 0.0502 | 0.0494 |
|  |  | External | 0.0300 | 0.0362 | **0.0373** | 0.0358 | 0.0225 | 0.0330 | 0.0347 | 0.0300 |
|  | Training Time [s] | Training | 6.87 | 66.64 | 244.20 | **2.74** | 14.68 | 14.26 | 32.24 | 606.70 |
| With 100 noisy features | AUROC | Internal | 0.6904 | 0.8541 | **0.8560** | 0.8469 | 0.8277 | 0.7652 | 0.8289 | 0.8134 |
|  |  | External | 0.6581 | 0.8059 | **0.8096** | 0.7937 | 0.7522 | 0.7162 | 0.7674 | 0.7372 |
|  | F1- Macro | Internal | 0.4026 | 0.4951 | 0.4773 | **0.5153** | 0.4416 | 0.4387 | 0.4704 | 0.4635 |
|  |  | External | 0.3842 | 0.4789 | 0.4608 | **0.5006** | 0.3769 | 0.4386 | 0.4696 | 0.4390 |
|  | MCC | Internal | 0.0220 | **0.0682** | 0.0596 | 0.0649 | 0.0439 | 0.0353 | 0.0508 | 0.0435 |
|  |  | External | 0.0126 | 0.0365 | **0.0368** | 0.0360 | 0.0224 | 0.0235 | 0.0323 | 0.0251 |
|  | Training Time [s] | Training | 15.24 | 223.51 | 809.45 | 12.82 | 102.85 | **6.38** | 19.72 | 297.97 |
