# Supplementary material for: Edges are all you need: Potential of medical time series analysis on complete blood count data with graph neural networks
Source: PLoS One. 2025 Jul 8;20(7):e0327636. doi: 10.1371/journal.pone.0327636 (PMC12237013; doi:10.1371/journal.pone.0327636)
Supplement: S4 Table — (DOCX) [file pone.0327636.s004.docx]

**S4 Table | Reference values for blood parameters in complete blood count analysis. Note, that these values highly depend on various factors including the measurement device, sex, age, and genetics.**

|  | Red blood cells [Gpt/l] | Hemoglobin [mmol/l] | White blood cells [Gpt/l] | Mean corpuscular volume [fl] | Platelets [Gpt/l] |
| --- | --- | --- | --- | --- | --- |
| **Lower bound** | 4.1 | 7.14 | 3.7 | 80 | 137 |
| **Upper bound** | 5.9 | 11.05 | 11.5 | 96 | 443 |
| **Reference** | Gulati et al.[1] | Nebe et al.[2] | Nebe et al.[2] | Nebe et al.[2] | Gulati et al.[1] |

**References**

**1**. Gulati GL, Hyun BH. The automated CBC. A current perspective. Hematol Oncol Clin North Am. 1994; 8:593–603.

**2**. Nebe T, Bentzien F, Bruegel M, Fiedler GM, Gutensohn K, Heimpel H, et al. Multizentrische Ermittlung von Referenzbereichen für Parameter des maschinellen Blutbildes/Multicentric Determination of Reference Ranges for Automated Blood Counts. LaboratoriumsMedizin. 2011; 35:3–28. doi: 10.1515/JLM.2011.004.
