# Supplementary material for: Edges are all you need: Potential of medical time series analysis on complete blood count data with graph neural networks
Source: PLoS One. 2025 Jul 8;20(7):e0327636. doi: 10.1371/journal.pone.0327636 (PMC12237013; doi:10.1371/journal.pone.0327636)
Supplement: S5 Table — (DOCX) [file pone.0327636.s005.docx]

**S5 Table | Attention weights of the last layer of the trained graph attention networks on each graph (directed graph, reverse directed graph and undirected graph). Each attention weight can vary between 0 (no influence) and 1 (high influence). We summarized all edges based on their source and target node label.**

|  | Directed graph | | | | Reverse directed graph | | | | Undirected graph | | | |
| --- | --- | --- | --- | --- | --- | --- | --- | --- | --- | --- | --- | --- |
|  | **Control to Control** | **Sepsis to Control** | **Control to Sepsis** | **Sepsis to Sepsis** | **Control to Control** | **Sepsis to Control** | **Control to Sepsis** | **Sepsis to Sepsis** | **Control to Control** | **Sepsis to Control** | **Control to Sepsis** | **Sepsis to Sepsis** |
| **Number of edges** | 7,245,987 | 0 | 10,691 | 2,533 | 7,245,987 | 10,691 | 0 | 2,533 | 12,675,003 | 10,691 | 10,691 | 2,602 |
| **Mean** | 0.251 | 0.000 | 0.054 | 0.747 | 0.251 | 0.001 | 0.000 | 0.973 | 0.143 | 0.056 | 0.057 | 0.711 |
| **Standard deviation** | 0.317 | 0.000 | 0.089 | 0.372 | 0.418 | 0.023 | 0.000 | 0.160 | 0.219 | 0.087 | 0.090 | 0.387 |
| **Minimum** | 0.000 | 0.000 | 0.000 | 0.000 | 0.000 | 0.000 | 0.000 | 0.000 | 0.000 | 0.001 | 0.001 | 0.002 |
| **25% quantile** | 0.035 | 0.000 | 0.008 | 0.381 | 0.000 | 0.000 | 0.000 | 1.000 | 0.025 | 0.009 | 0.010 | 0.320 |
| **50%- quantile** | 0.110 | 0.000 | 0.020 | 1.000 | 0.000 | 0.000 | 0.000 | 1.000 | 0.063 | 0.023 | 0.023 | 1.000 |
| **75%- quantile** | 0.318 | 0.000 | 0.058 | 1.000 | 0.417 | 0.000 | 0.000 | 1.000 | 0.154 | 0.062 | 0.062 | 1.000 |
| **Maximum** | 1.000 | 0.000 | 0.987 | 1.000 | 1.000 | 1.000 | 0.000 | 1.000 | 1.000 | 0.959 | 0.846 | 1.000 |
