# Supplementary material for: Edges are all you need: Potential of medical time series analysis on complete blood count data with graph neural networks
Source: PLoS One. 2025 Jul 8;20(7):e0327636. doi: 10.1371/journal.pone.0327636 (PMC12237013; doi:10.1371/journal.pone.0327636)
Supplement: Attachment — Submitted filename: Review-PlosOne.pdf [file pone.0327636.s006.pdf]

This manuscript titled “Edges are all you need: Potential of Medical Time Series 2 Analysis with Graph Neural Networks” introduced an approach for incorporating time-series clinical diagnosis data efficiently, and showed that graph neural networks (GNNs) provide a better alternative than traditional machine learning (ML) algorithms. While I think this work is very well-written and well explained, the novelty and impact of this work falls short for acceptance in PLOS One. Therefore, I reject this manuscript. I addressed the concerns below:

1. **Novelty issue:** The main objective of this paper and the graph neural network based approach is not new for clinical data. Several previous works have already applied GNN on clinical data. Some of the previous works are listed below [1 - 4]. Moreover, the feature importance calculation part is also not the invention of the authors, as their mentioned process falls under a special form of ablation study highly done in GNN papers. Furthermore, the GNNs they used to evaluate performance are also not developed by the authors. Under these circumstances, I believe this work has not been able to meet the novelty criteria for PLOS One.

[1] Wang, Yanan, Yu Guang Wang, Changyuan Hu, Ming Li, Yanan Fan, Nina Otter, Ikuan Sam et al. "Cell graph neural networks enable the precise prediction of patient survival in gastric cancer." NPJ precision oncology 6, no. 1 (2022): 45.

[2] Sun, Zhenchao, Hongzhi Yin, Hongxu Chen, Tong Chen, Lizhen Cui, and Fan Yang. "Disease prediction via graph neural networks." IEEE Journal of Biomedical and Health Informatics 25, no. 3 (2020): 818-826.

[3] Gao, Jianliang, Tengfei Lyu, Fan Xiong, Jianxin Wang, Weimao Ke, and Zhao Li. "Predicting the survival of cancer patients with multimodal graph neural network." IEEE/ACM Transactions on Computational Biology and Bioinformatics 19, no. 2 (2021): 699-709.

[4] Li, Yang, Buyue Qian, Xianli Zhang, and Hui Liu. "Graph neural network-based diagnosis prediction." Big data 8, no. 5 (2020): 379-390.

2. **Work amount issue:** The authors only evaluated performances for one dataset which is quite inadequate for evaluating performances across different types of data. Without showing that their approach generalizes for multiple types of data, this work cannot be accepted.
3. **Results issue:** According to the authors comments, if incorporating time series data improves performance for these types of data, than better alternative are recurrent neural networks (RNNs) and Transformers. But no comparison was shown of the GNNs with these types of models. GNNs are more suited for data that has natural graph-like structures (e.g., crystals, proteins, molecules, RNA,

etc.). So, without comparing it with at least an LSTM model (RNN) [1], I cannot understand the impact of this work.

[1] Hochreiter, Sepp, and Jürgen Schmidhuber. "Long short-term memory." Neural computation 9, no. 8 (1997): 1735-1780.

4. "*However, this information is mostly neglected by state-of-the-art machine learning algorithms*" - you need to cite some works.
5. "*Furthermore, GNNs and other complex machine learning algorithms (e.g., XGBoost) are often treated as black-boxes limiting their interpretability and transparency which is essential for medical applications.*" - this work also does not address this issue. The feature importance calculation does not address this issue as this refers to the interpretability of the neural network itself. For example, what sort of information the output of each layer (latent space) bears.
6. What is the validity of synthetic data generated? No explanation was provided.
7. "*The reason for this similar performance is that the nodes of complete blood counts only sample information from similar node blood count measurements (measurement-centric graphs).*" - not a strong reason, need to describe with respect to the GNN architecture.
8. No details on the GNN algorithms used. The readers need to know the scientific reasons why GNN is performing better than traditional ML models. The authors need to explain why particular GNN architecture performed better, and why particular GNN architecture performed worse. Because this work can be iteratively improved, but if they don't delve into the GNN architecture, this becomes very hard to improve logically.
