# Supplementary material for: Edges are all you need: Potential of medical time series analysis on complete blood count data with graph neural networks
Source: PLoS One. 2025 Jul 8;20(7):e0327636. doi: 10.1371/journal.pone.0327636 (PMC12237013; doi:10.1371/journal.pone.0327636)
Supplement: Attachment — Submitted filename: JNCA_D_24_00921_R1_reviewer.pdf [file pone.0327636.s008.pdf]

1 Original Submission

Manuscript Number: PONE-D-24-06777R1
Edges are all you need: Potential of Medical Time Series Analysis on Complete Blood Count Data with Graph Neural Networks.
Journal: PLOS ONE
This manuscript highlights that the approach of developing algorithms for clinical diagnosis using standard machine learning algorithms is less efficient than using Graph Neural Networks (GNN), as the data are interconnected and present time series. The approach presented here is based on clinical data graphs. The main goal of this study is to evaluate when GNNs represent a valuable alternative for analyzing large clinical data from the clinical routine on the example of Complete Blood Count Data. For incorporating multiple measurements of the same patient, author construct several patient-centric graphs where the node represents standard normalized complete blood counts, and edges represent connections from previous to following measurements.
The article lacks coherence between the introduction and the conclusion and does not delve into the GNN algorithms used. It also fails to provide an explanation of why some GNN algorithms perform better than others, nor does it discuss the implemented architecture. This significantly reduces its scientific value. The absence of such crucial details makes it difficult for the reader to fully understand the work. Due to these reasons, as well as those outlined in the major comments, I believe the article is not suitable for publication in the journal.

2 Recommendation

Reject

3 Major Comments

There should be an explanation of why GNNs algorithms have heterogeneous nature of the attributes associated with different types of nodes and why represent nodes in a lower-dimensional space.
Dataset size issue: the tests were conducted on only one dataset. It is necessary to consider the validity and homogeneity of the data, how the data are generated and collected in order to obtain more information. E.g. Are they from various countries? Are the patients taking medications or not? Are there children? One correlate point concerns bias: when dealing with clinical trials data, in order to correctly classify the examples, it is necessary to have a larger number of datasets for training neural networks without consequent crystallizing of stereotypes. I appreciated that there are Supplemental Material and the legends in the figures, but I suggest the authors add a graphical sketch to illustrate the workflow of your study to improve the reader's understanding.

- p2 line 29. In the introduction section, after describing the examples of the types of graphs, it was not specified which ones are being used.
- p2 line 37. In this manuscript which is the architecture?
- p2 line 42. You should be use extended form before using the "AUROC" acronym.
- p3 line 46 There is no explanation of what the complete blood count data are; I would suggest including a parenthetical explanation.
- p6 lines 120/121 in "we compared classification" and "we evaluated" the references to the figures are missing. In results section, additional information on the results obtained from the prediction could also be included.
- p7. The GNNs algorithms used are not specified.
- p24. in Fig I, node 4 is repeated twice.
- In section 4, please specify the figures/tables associated with the results being referenced.

4 Minor Comments

- p6 line 128 In results section to facilitate reading, details could be added in parentheses "internal and external test set".
- in general, double-check that some definitions correspond with those provided in the figure, e.g. p6- 2.5 reverse(d) direct graph in Fig. 4E was reversed and p8- 3.3.
- p8. As for the plotted results, it is not specified which total features were used, or they could be included as reported in Section 2.4 and they are mentioned only at the end of the section in line 171. Line 169 The letter 'G' to the figures is missing. line 175 The references to the fig. 4 D and F are missing.
- p9 line 188 The reference to the table 1 is missing. Line 198 The reference to the figure 3 is missing
- p10 The reference to the table 2 is missing. line 205 replace GNN in GAT and the reference to the figure 4 is missing.
- Why in supplementary materials in the violin plots you not visualized the feature "sex" since it is a categorical feature (i.e., either "male" and "female" were reported)?
