# Supplementary material for: Edges are all you need: Potential of medical time series analysis on complete blood count data with graph neural networks
Source: PLoS One. 2025 Jul 8;20(7):e0327636. doi: 10.1371/journal.pone.0327636 (PMC12237013; doi:10.1371/journal.pone.0327636)
Supplement: Attachment — Submitted filename: Response To Reviewers.docx [file pone.0327636.s009.docx]

**Reviewer 1**

**Thank you for your feedback. We hope that the extended results improved our manuscript.**

*The authors emphasize the important of making connections between samples.
However, the experimental results do not show that it is more effective to analyze samples from the perspective of graphs. There are some concerns.*

*#R1_1: When analyzing similar-graphs, GNNs are comparable in performance to other algorithms, but less robust and longer training time;*

**#R1_1 Answer: Yes, that is why we would like to emphasize not using GNNs for similarity-based graph structures. However, we can achieve improvements with GNNs on patient-centric graphs (see #R1_2).**

*#R1_2: When analyzing patient-centric graphs, it seems more like the attention mechanism works, not graph struture. I do not think other GNNs (i.e. GCN) can achieve the same high performance. More experiments may be needed here.*

**#R1_2 Answer: We performed further experiments of GNNs (GraphSAGE and GCN) on patient-centric graphs. The performance on GraphSAGE was comparable to the performance of GAT indicating that the attention mechanism is not necessary for the observed improvements. GCN has a bit lower performance on directed and undirected graph structures which might be the consequence of the symmetric normalization and the linear transformation before the message-propagation (e.g., GraphSAGE first propagate and then transforms the data). However, on the reversed-directed graphs the performance was similar to the GAT (see #R1_2).**

*#R1_3: In addition, I wonder how the attributes of various types of nodes in heterogeneous similarity graphs are defined, since the attributes in homogeneous similarity graphs are used as nodes.*

**#R1_3 Answer: We tried to clarify the explanation more (see #R1_3).**

*#R1_4: Despite these concerns, I think it makes sense to use GNNs to analyze medical data. The authors may need to further explore the way they consturct the graphs, any why GNNs are more efficient.*

**#R1_4 Answer: Our main objective was to improve clinical decision support systems with clinical routine data like complete blood count data with algorithms best suited for the underlying data. We highlighted this focus now better in our title. In this process, we figured out that incorporating the information from time series information (patient-centric graphs) was much more promising than considering the complete blood count measurements independent of the patient (e.g., similarity graphs).**

**Reviewer 2**

**Thank you for your comprehensive feedback on our work. We hope that the updated changes and extended results improved the manuscript.**

*This manuscript titled “Edges are all you need: Potential of Medical Time Series 2 Analysis with Graph Neural Networks” introduced an approach for incorporating time-series clinical diagnosis data efficiently, and showed that graph neural networks (GNNs) provide a better alternative than traditional machine learning (ML) algorithms. While I think this work is very well-written and well explained, the novelty and impact of this work falls short for acceptance in PLOS One. Therefore, I reject this manuscript. I addressed the concerns below:

#R2_1: 1. Novelty issue: The main objective of this paper and the graph neural network based approach is not new for clinical data. Several previous works have already applied GNN on clinical data. Some of the previous works are listed below [1 - 4]. Moreover, the feature importance calculation part is also not the invention of the authors, as their mentioned process falls under a special form of ablation study highly done in GNN papers. Furthermore, the GNNs they used to evaluate performance are also not developed by the authors. Under these circumstances, I believe this work has not been able to meet the novelty criteria for PLOS One.

[1] Wang, Yanan, Yu Guang Wang, Changyuan Hu, Ming Li, Yanan Fan, Nina Otter, Ikuan Sam et al. "Cell graph neural networks enable the precise prediction of patient survival in gastric cancer." NPJ precision oncology 6, no. 1 (2022): 45.
[2] Sun, Zhenchao, Hongzhi Yin, Hongxu Chen, Tong Chen, Lizhen Cui, and Fan Yang. "Disease prediction via graph neural networks." IEEE Journal of Biomedical and Health Informatics 25, no. 3 (2020): 818-826.
[3] Gao, Jianliang, Tengfei Lyu, Fan Xiong, Jianxin Wang, Weimao Ke, and Zhao Li. "Predicting the survival of cancer patients with multimodal graph neural network." IEEE/ACM Transactions on Computational Biology and Bioinformatics 19, no. 2 (2021): 699-709.
[4] Li, Yang, Buyue Qian, Xianli Zhang, and Hui Liu. "Graph neural network-based diagnosis prediction." Big data 8, no. 5 (2020): 379-390.*
**#R2_1 Answer: *We included the references in our introduction section and have rewritten the sections and clarified our main objectives (see #R2_1). Our main objective is to improve clinical decision support systems with clinical routine data like complete blood count data with algorithms best suited for the underlying data (time series data of different length). Regardless of this objective, the novelty criteria does not apply to PLOS ONE as indicated on their homepage: „The world’s first multidisciplinary Open Access journal, PLOS ONE accepts scientifically rigorous research, regardless of novelty.“ (see https://everyone.plos.org/about-plos-one/)***

*#R2_2: 2. Work amount issue: The authors only evaluated performances for one dataset which is quite inadequate for evaluating performances across different types of data. Without showing that their approach generalizes for multiple types of data, this work cannot be accepted.*

**#R2_2 Answer: We never intended nor described to evaluate the performance across multiple data types. Our objective was to evaluate the classification performance on complete blood count data from clinical routine (see answer #R2_1). An external validation of our results was provided with an external validation dataset from a different tertiary care center.**

*#R2_3 3. Results issue: According to the authors comments, if incorporating time series data improves performance for these types of data, than better alternative are recurrent neural networks (RNNs) and Transformers. But no comparison was shown of the GNNs with these types of models. GNNs are more suited for data that has natural graph-like structures (e.g., crystals, proteins, molecules, RNA, etc.). So, without comparing it with at least an LSTM model (RNN) [1], I cannot understand the impact of this work.*

*[1] Hochreiter, Sepp, and Jürgen Schmidhuber. "Long short-term memory." Neural computation 9, no. 8 (1997): 1735-1780.*

**#R2_3 Answer: We introduced an explicit decoder-only transformer architecture, 1D-CNN, LSTM and Bi-LSTM for the sake of completeness (see #R2_3). In contrast to the GNN architecture, these architectures (at least the Transformer and 1D-CNN, and for efficient batching also LSTM architectures) required an additional padding and masking step which exacerbates their usage for time series of different lengths.**

*#R2_4: “However, this information is mostly neglected by state-of-the-art machine learning algorithms" - you need to cite some works.*

***#R2_4 Answer: We clarified the statement (see #R2_4).***

*#R2_5 5. “Furthermore, GNNs and other complex machine learning algorithms (e.g., XGBoost) are often treated as black-boxes limiting their interpretability and transparency which is essential for medical applications.” - this work also does not address this issue. The feature importance calculation does not address this issue as this refers to the interpretability of the neural network itself. For example, what sort of information the output of each layer (latent space) bears.*

*#R2_6 6. What is the validity of synthetic data generated? No explanation was provided.*

**#R2_5+6 Answer: While writing the manuscript, we unfortunately were not aware of the existence of partial dependence plots (**[**https://hastie.su.domains/ElemStatLearn/**](https://hastie.su.domains/ElemStatLearn/)**) which were precisely our goal (i.e., investigating prediction changes based on different feature values). Therefore, we changed our methodology to the partial dependence plots and plotted the average prediction values across different grid values between the 5% and 95% percentile (see #R2_5+6). Thereby, we can directly see the influence of different values of individual features to the overall prediction of a specific complete blood count measurement.**

*#R2_7+8: 7. “The reason for this similar performance is that the nodes of complete blood counts only sample information from similar node blood count measurements (measurement-centric graphs).” - not a strong reason, need to describe with respect to the GNN architecture.*

*8. No details on the GNN algorithms used. The readers need to know the scientific reasons why GNN is performing better than traditional ML models. The authors need to explain why particular GNN architecture performed better, and why particular GNN architecture performed worse. Because this work can be iteratively improved, but if they don’t delve into the GNN architecture, this becomes very hard to improve logically.*

**#R2_7+8 Answer: We added further information as explanations including the individual architectures of the investigated GNNs (see #R2_7+8)**
