# Supplementary material for: Edges are all you need: Potential of medical time series analysis on complete blood count data with graph neural networks
Source: PLoS One. 2025 Jul 8;20(7):e0327636. doi: 10.1371/journal.pone.0327636 (PMC12237013; doi:10.1371/journal.pone.0327636)
Supplement: Attachment — Submitted filename: ResponseToReviewers_RH.docx [file pone.0327636.s010.docx]

**We sincerely thank the editor and the reviewers for their thoughtful and constructive feedback, which has helped us improve the quality and clarity of our work. We greatly appreciate the time and effort invested in reviewing our manuscript and providing detailed comments and suggestions. Below, we address each comment point by point and describe the corresponding changes made to the manuscript starting with Additional Editor Comments. We are confident that these revisions have significantly strengthened the work and hope they meet the reviewer’s expectations.**

**Additional Editor Comments**

*The second round of reviews is now complete. My opinion is that the manuscript must undergo a major revision before considering for publication in PLOS ONE.
What emerges is that the manuscript lacks a unitary style. In addition, the abstract and the introduction should (at least) mention all the algorithms used in the experiments. In this respect, the abstract and the introduction fail in presenting the research.*

**We revised the style and writing according to the reviewers’ suggestions. We added the GNNs to the abstract and introduction (see Editor request).**

*[Note: HTML markup is below. Please do not edit.]*

*Reviewers' comments:*

*Reviewer's Responses to Questions*

***Comments to the Author***

*1. If the authors have adequately addressed your comments raised in a previous round of review and you feel that this manuscript is now acceptable for publication, you may indicate that here to bypass the “Comments to the Author” section, enter your conflict of interest statement in the “Confidential to Editor” section, and submit your "Accept" recommendation.*

*Reviewer #3: (No Response)*

*Reviewer #4: All comments have been addressed*

*2. Is the manuscript technically sound, and do the data support the conclusions?

The manuscript must describe a technically sound piece of scientific research with data that supports the conclusions. Experiments must have been conducted rigorously, with appropriate controls, replication, and sample sizes. The conclusions must be drawn appropriately based on the data presented.*

*Reviewer #3: Yes*

*Reviewer #4: No*

*3. Has the statistical analysis been performed appropriately and rigorously?*

*Reviewer #3: Yes*

*Reviewer #4: Yes*

*4. Have the authors made all data underlying the findings in their manuscript fully available?*

*The* [*PLOS Data policy*](http://www.plosone.org/static/policies.action#sharing) *requires authors to make all data underlying the findings described in their manuscript fully available without restriction, with rare exception (please refer to the Data Availability Statement in the manuscript PDF file). The data should be provided as part of the manuscript or its supporting information, or deposited to a public repository. For example, in addition to summary statistics, the data points behind means, medians and variance measures should be available. If there are restrictions on publicly sharing data—e.g. participant privacy or use of data from a third party—those must be specified.*

*Reviewer #3: Yes*

*Reviewer #4: Yes*

*5. Is the manuscript presented in an intelligible fashion and written in standard English?

PLOS ONE does not copyedit accepted manuscripts, so the language in submitted articles must be clear, correct, and unambiguous. Any typographical or grammatical errors should be corrected at revision, so please note any specific errors here.*

*Reviewer #3: Yes*

*Reviewer #4: Yes*

*6. Review Comments to the Author*

*Please use the space provided to explain your answers to the questions above. You may also include additional comments for the author, including concerns about dual publication, research ethics, or publication ethics. (Please upload your review as an attachment if it exceeds 20,000 characters)*

***Reviewer #3***

*The manuscript presents an original study that applies Graph Neural Networks (GNNs) to predict sepsis using Complete Blood Count (CBC) data. This is an interesting and promising approach, but the paper has several areas that require improvement. While the second half of the paper addresses many initial issues, there are gaps in clarity, the experimental setup, and interpretability that need to be tackled before the manuscript can be considered for publication.*

*#R3_1: Originality*

*The application of GNNs to patient-centric graphs for sepsis detection is novel and contributes to ongoing research into machine learning in healthcare. However, the work overlaps with previous studies like Chen et al. (Eur Rev Med Pharmacol Sci 2021; 25 (14): 4693-4701, DOI: 10.26355/eurrev_202107_26380),  which used GNNs for pediatric sepsis. A more explicit comparison with this and similar works, along with a justification of the broader patient population, would strengthen the claim of originality.*
**Answer #R3_1: We included the reference and highlighted that they classified pediatric sepsis instead of sepsis in adults, only used similarity graphs and incorporated multiple laboratory tests instead of using only data from the clinical routine like complete blood count data (“[GNNs] already showed promising results for predicting pediatric sepsis based on several groups of laboratory tests (e.g., medical history and serological tests) using similarity graphs [17].”see #R3_1).**

*#R3_2: Clinical Relevance*

*The selection of CBC parameters is reasonable, but the relevance of each feature to sepsis prediction should be better explained. For readers unfamiliar with clinical data, it would be helpful to briefly introduce why these specific markers are key indicators of sepsis and to support this with appropriate references.*

**Answer #R3_2: We added a supplementary note to explain the functions of individual blood parameters and their potential relevance for sepsis. A text reference for the supplementary note was added in the manuscript (“The functions and the potential relevance of the individual blood parameters for sepsis is discussed in the supplement (Supplementary Note 1)”, see #R3_2).**

*#R3_3: Ethical Considerations*

*The manuscript states that ethical approval was not applicable due to the anonymization of the data. However, further elaboration is necessary regarding how the privacy of patient data was ensured. Data privacy is particularly sensitive in healthcare applications, and a brief explanation of how the dataset was anonymized would help meet ethical transparency standards.*

**Answer #R3_3: We added ethical approvals from the original study of Steinbach et al. (“The Ethics Committee at the Leipzig University Faculty of Medicine approved the initial study from Steinbach et al.**

**[26] (reference number: 214/18-ek). The study was published in accordance with the transparent Reporting of a multivariable prediction model for Individual Prognosis or Diagnosis (TRIPOD) statement. This study is only re-evaluating the dataset from Steinbach et al. [26] by evaluating GNNs and incorporating time-series information“, see #R3_3).**

*Evaluation of Paper Sections*

*#R3_4:• Abstract*

*The abstract is concise but could be clearer. For instance, it mentions AUROC as the primary evaluation metric but doesn’t justify why this metric was chosen over others like the F1 score, which is more commonly use. A brief justification for focusing on AUROC would be helpful. Additionally, more details about the graph structure (why it was used over simpler models) and how GNNs handle time-series data would improve the clarity.*

**Answer #R3_4: We added respective details in the abstract (“Methods: In this study, we evaluated the performance and time consumption of several GNNs (e.g., Graph Attention Networks) on similarity graphs compared to simpler, state-of-the-art machine learning algorithms (e.g., XGBoost) on the classification of sepsis from blood count data as well as the importance and slope of each feature for the final classification. Additionally, we connected complete blood count samples of the same patient based on their measured time (patient-centric graphs) to incorporate time series information in the GNNs. As our main evaluation metric we used the Area Under Receiver Operating Curve (AUROC) to have a threshold independent metric that can handle class imbalance.**

**Results and Conclusion: Standard GNNs on evaluated similarity-graphs achieved an Area Under Receiver Operating Curve (AUROC)[...]”, see #****R3_4).**

*#R3_5:• Introduction*

*The introduction is somewhat lacking in depth, particularly concerning the clinical background of sepsis and the rationale for using GNNs. A more detailed explanation of why GNNs are suited for sepsis prediction, compared to simpler methods, would benefit the reader. The discussion of DeepWalk and Node2Vec compares them to GNNs, implying they are machine learning algorithms. These methods are more accurately described as graph embedding techniques used for feature representation, not prediction. This section would benefit from clarification and more technical precision. The introduction should also be expanded to give a clearer description of how the graph structure was defined. The definition of nodes and edges is somewhat imprecise and could confuse readers unfamiliar with graph theory terminology. For example, terms like "vertices" and "links" are interchangeable with "nodes" and "edges," but their use should be consistent.*

**Answer #R3_5: We corrected our definition of a graph and clarified that DeepWalk and node2vec are embedding techniques. Then, we added more details regarding the clinical background of sepsis. Finally, we added more details on how we defined the graph structures and the rationale behind using GNNs (“A graph G is a non-empty finite set of elements called nodes V(G) and finite set E(G) of distinct unordered pairs of distinct elements of V(G) called edges**

**[6]. [...]GNNs have the advantage that they can utilize attached features and parallelize computations on modern hardware (e.g., GPUs) in contrast to using embedding techniques like DeepWalk [15] or Node2Vec [16]. […] Sepsis is a life-threatening organ dysfunction caused by a dysregulated immune response to an infection**

**[28]. The inflammatory response is driven through the release of cytokines from neutrophil granulocytes and macrophages. Blood parameters like white blood cells, red blood cells, platelets, hemoglobin and mean corpuscular volume might serve as easily available indicators for sepsis [26] (Supplementary Note 1). ”, see #R3_5).**

*#R3_6: • Methods*

*The methods section provides a detailed explanation of the experimental setup but lacks justification for some key decisions. For instance, why were tree-based algorithms chosen as baselines? Further, the reasoning behind certain hyperparameter choices, such as the number of epochs and learning rate, is unclear. More justification for selecting GraphSAGE as the representative GNN is necessary: were other GNN architectures considered, and if so, why was GraphSAGE chosen as the final model?*

**Answer #R3_6: We used tree-based and non-tree based (e.g., logistic regression and a neural network) as benchmarks to get a comprehensive overview of the** **performance evaluation across several algorithms. We also added justifications for chosen hyperparameters and why we chose GraphSAGE as final GNN model for the interpretability (“As benchmarks, we evaluated the performance (AUROC, F1- Macro Score, and MCC) on tree-based and non-tree-based algorithms (Fig. 1 C) to get a comprehensive performance evaluation across several algorithms. […] The high epoch number in combination with a low learning rate should prevent under-fitting. Early-stopping on a separate validation set was used to prevent over-fitting. […] GraphSAGE was chosen as the final model since it achieved a reliable classification performance on the homogeneous and heterogeneous similarity graphs.”, see #R3_6).**

*#R3_7: Results*

*The results section is one of the paper’s strengths, providing a comprehensive evaluation of the models and a comparison between different machine learning approaches. However, the choice of AUROC as the primary metric remains problematic. The authors do mention F1 later in the paper but should provide more justification for emphasizing AUROC initially. The partial dependence plots in the results are well-executed, but the explanation of how the direction of individual features (e.g., an increase in white blood cell count) affects predictions could be clearer. While the paper mentions this limitation, providing more interpretability around feature impact is crucial, particularly in clinical settings where decisions depend on understanding how individual factors contribute to risk.*

**Answer #R3_7: We added justifications why chose AUROC as primary evaluation metric (“In the following, we will mainly focus on AUROC as the primary evaluation metric to have a threshold-independent evaluation metric that can also incorporate the high class imbalance. By assessing model performance across different thresholds, AUROC enables clinicians to fine-tune the sensitivity and specificity of sepsis detection according to their needs, minimizing both the risk of missing septic patients and the potential harm from overdiagnosis, such as unnecessary antibiotic treatments.”, see #R3_7). Additionally, we provided clearer explanations for the partial dependence plots (“Specifically, that means older people with higher white blood cell counts, red blood cell counts and increased corpuscular volume have higher sepsis probabilities according to the trained models. White blood cells had a minimum of around 4 – 8 Gpt/l (Giga- particles per liter) for most algorithms (Fig. 3 D) that indicates a physiological white blood cell count at this range. In contrast to the positive gradient, we observed a negative gradient for platelets for all algorithms (Fig. 3 G), indicating decreased sepsis risk for rising values according to the models. Specifically, this means that patients with lower platelets (thrombocytopenia) have a higher sepsis probability according to the trained models. Tree-based algorithms (Decision Tree, RUSBoost, Random Forest, XGBoost) do not depend (gradient near zero) on the features hemoglobin (Fig. 3 C) and red blood cells (Fig 3 E) in contrast to non-tree-based algorithms (Logistic Regression, Neural Network, Homogeneous GNN, Heterogeneous GNN). The latter ones showed an increased sepsis probability for low hemoglobin levels (anemia) (Fig. 3 C). Finally, the feature “sex” is nearly irrelevant for all algorithms (i.e., low gradient), i.e., the sepsis probability does not significantly depend on sex (Fig. 3 B). ”, see #R3_7). In terms of interpretability, we are actively developing a novel graph learning framework aimed at significantly improving interpretability, which remains a focus of our ongoing work.**

*#R_3_8: • Discussion*

*The discussion section does a good job of addressing the limitations of the study, including bias in the dataset and computational challenges. However, the paper would benefit from a more detailed analysis of the computational complexity of GNNs, which are known to be resource-intensive. Were any optimizations considered, such as using more efficient GNN architectures or limiting the number of layers to reduce computational overhead?*

**Answer #R3_8: We added a description for the general time complexity of GNNs (“In general, the computational complexity of a single convolution layer of a GNN is O(VFF’ + EF’), where V represents the number of nodes in the graph, E the number of edges in the graph, F the number of input features and F’ the hidden dimension or output features for the convolution [8,43]. However, the computational complexity differs across different architectures, depending on factors like the number of attention heads (e.g., in GAT and GATv2), and used multi-layer perceptrons (e.g., in GIN). ”see #R3_8). The number of layers was already quite low with only two layers. One layer was evaluated in initial experiments but showed a significantly lower classification performance. In terms of reducing computational overhead, as stated above we are currently working on a new graph learning framework that reduces computational complexity compared to Graph Neural Networks (GNNs) while significantly enhancing interpretability of results.**

*• Data and Code Availability*

*The transparency in making the code and data publicly available on GitHub and Zenodo is commendable and aligns with best practices in reproducibility. This adherence to open science is a strength of the paper, ensuring that the study can be replicated and verified by other researchers.*

*• Appendix*

*The appendix provides valuable supplementary materials, including tables, plots, and diagrams that enhance the main text. These materials clarify several points that are less detailed in the main sections.*

*Recommendation
The study presents a novel application of GNNs to sepsis prediction and shows strong experimental work. However, the manuscript requires revisions to improve its clarity and justification of choices in both methodology and evaluation metrics. I recommend a minor revision to address these issues before it can be considered for publication in PLOS ONE.*

**Reviewer #4**

*The article lacks coherence between the introduction and the conclusion and does not delve into the GNN algorithms used. It also fails to provide an explanation of why some GNN algorithms perform better than others, nor does it discuss the implemented architecture. This significantly reduces its scientific value. The absence of such crucial details makes it difficult for the reader to fully understand the work. Due to these reasons, as well as those outlined in the major comments (see attached file), I believe the article is not suitable for publication in the journal.*

*This manuscript highlights that the approach of developing algorithms for clinical diagnosis using standard machine learning algorithms is less efficient than using Graph Neural Networks (GNN), as the data are interconnected and present time series. The approach presented here is based on clinical data graphs. The main goal of this study is to evaluate when GNNs represent a valuable alter- native for analyzing large clinical data from the clinical routine on the example of Complete Blood Count Data. For incorporating multiple measurements of the same patient, author construct several patient-centric graphs where the node represents standard normalized complete blood counts, and edges represent connections from previous to following measurements.*

*#R4_1: The article lacks coherence between the introduction and the conclusion and does not delve into the GNN algorithms used. It also fails to provide an explanation of why some GNN algorithms perform better than others, nor does it discuss the implemented architecture. This significantly reduces its scientific value. The absence of such crucial details makes it difficult for the reader to fully understand the work. Due to these reasons, as well as those outlined in the major comments, I believe the article is not suitable for publication in the journal.*

**Answer #R4_1: The primary objective of our work is to assess the applicability of various machine learning algorithms to clinical routine data, specifically complete blood count data. Additionally, we aimed to explore the interpretability of these algorithms, investigating individual predictions through techniques such as partial dependence plots. We highlighted the explanations for different architectures and potential performance differences and discussions about implemented architectures (see #R4_1).**

1 ***Recommendation***

*Reject*

2 ***Major Comments***

*#R4_2: There should be an explanation of why GNNs algorithms have heterogeneous nature of the attributes associated with different types of nodes and why represent nodes in a lower-dimensional space.*

**Answer #R4_2: This seems to be a misunderstanding. We suspect that you refer to our heterogeneous graph structures. With this graph structure, we only wanted to evaluate whether this indirect similarity of complete blood count measurements could increase the performance compared to homogeneous graphs. However, since the performance did not increase, we suspect that generally including information from similar measurement does not yield much value.**

*#R4_3: Dataset size issue: the tests were conducted on only one dataset. It is necessary to consider the validity and homogeneity of the data, how the data are generated and collected in order to obtain more information. E.g. Are they from various countries? Are the patients taking medications or not? Are there children? One correlate point concerns bias: when dealing with clinical trials data, in order to correctly classify the examples, it is necessary to have a larger number of datasets for training neural networks without consequent crystallizing of stereo- types. I appreciated that there are Supplemental Material and the legends in the figures, but I suggest the authors add a graphical sketch to illustrate the workflow of your study to improve the reader’s understanding.*

**Answer #R4_3: We thank you for this concern but are quite optimistic that most of your missing information is stated in the paper. Regarding the dataset size: We used a dataset with over 1,000,000 complete count data, given the small number of features (seven), this is a very large dataset. You are correct that we only investigated one dataset. However, this dataset already includes independent data from different tertiary care centers (Leipzig & Greifswald) in Germany.**

**To test the validity and homogeneity of data, an external dataset (complete blood count data from Greifswald) from a different German tertiary center was used.**

**Information regarding the data generation are listed in the original submission of Steinbach et al.. All data were from Germany as indicated in “non-intensive care units from German tertiary care center in Leipzig (internal dataset) and Greifswald (external dataset) between January 2014 and December 2021” (see #R4_3). Medication information was not available. All patients were adults (see violin plot for age in the supplementary). The dataset has a huge size with over 1,000,000 data points which is also listed in Figure 1. The workflow is also listed in Figure 1 (see #R4_3).**

- *#R4_4: p2 line 29. In the introduction section, after describing the examples of the types of graphs, it was not specified which ones are being used.*

**Answer #R4_4: The goal of the introduction is to present the research topic, its significance and to provide a clear overview of the study’s purpose. However, the methodology is designed to describe the research design and analysis procedure. Therefore, details regarding the research design can be found in the methodology (see #R4_4).**

- *#R4_5: p2 line 37. In this manuscript which is the architecture?*

**Answer #R4_5: This is the general scheme of message passing in GNNs. There exist different architectures which is described in the next sentence.**

- *#R4_6: p2 line 42. You should be use extended form before using the ”AUROC” acronym.*

**Answer #R4_6: Thank you for mentioning this. We revised this (“[…] Area under Receiver Operating Curve (AUROC) [...]” see #R4_6).**

- *#R4_7: p3 line 46 There is no explanation of what the complete blood count data are; I would suggest including a parenthetical explanation.*

**Answer #R4_7: We added further information (“[…] complete blood count (i.e., hemoglobin, red blood cells, white blood cells, platelets and mean corpuscular volume [...]”, see #R4_7).**

- *#R4_8: p6 lines 120/121 in ”we compared classification” and ”we evaluated” the references to the figures are missing. In results section, additional information on the results obtained from the prediction could also be included.*

**Answer #R4_8: We tried our best to find the section you provided but did not find anything. Page 6 also only contains information about the methodology. Still, we now mention the corresponding tables and figures at several more places in the paper.**

- *#R4_9: p7. The GNNs algorithms used are not specified.*

**Answer #R4_9: We added the GNN algorithms (“Therefore, we applied several GNNs (Graph SAGE[7], GAT[8], GATv2[32], GATv2[32], GIN[10], HGT[12], and HAN[11]) [...]” see #R4_9).**

- *#R4_10: p24. in Fig I, node 4 is repeated twice.*

**Answer #R4_10: Thanks for remarking this. We fixed this error (see #R4_10).**

- *#R4_11: In section 4, please specify the figures/tables associated with the results being referenced.*

**Answer #R4_11: We added this (see #R4_11).**

3 ***Minor Comments***
  - *#R4_12: p6 line 128 In results section to facilitate reading, details could be added in parentheses ”internal and external test set”.*

**Answer #R4_12: We did not find the respective section you meant.**

- *#R4_13: in general, double-check that some definitions correspond with those provided in the figure, e.g. p6- 2.5 reverse(d) direct graph in Fig. 4E was reversed and p8- 3.3.*

**Answer #R4_13: Thanks for noticing this flaw. We fixed this (see #R4_13).**

- *#R4_14: p8. As for the plotted results, it is not specified which total features were used, or they could be included as reported in Section 2.4 and they are mentioned only at the end of the section in line 171. Line 169 The letter ’G’ to the figures is missing. line 175 The references to the fig. 4 D and F are missing.*
- **Answer #R4_14: We suppose you investigated the old, deleted figure instead of the new revised figure. However, we added further references in the text for clarification (see #R4_14).**
- *#R4_15: p9 line 188 The reference to the table 1 is missing. Line 198 The reference to the figure 3 is missing*

**Answer #R4_15: We added the references.**

- *#R4_16: p10 The reference to the table 2 is missing. line 205 replace GNN in GAT and the reference to the figure 4 is missing.*

**Answer #R4_16: If you mean the sentence “Nevertheless, GNNs, the neural network and ensemble-based algorithms could outperform shallow algorithms (decision tree and logistic regression) due to more expressive representations of the underlying information”, we meant GNNs on similarity graphs not on the patient-centric graphs. We specified this now for clarification (“Nevertheless, GNNs on similarity graphs, the neural network and ensemble-based algorithms could outperform shallow algorithms (decision tree and logistic regression) due to more expressive representations of the underlying information (Table 1).”, see#R4_15) and added references (see #R4_15). If this was not your concern, we would like to get clarification.**

- *#R4_17: Why in supplementary materials in the violin plots you not visualized the feature “sex” since it is a categorical feature (i.e., either “male” and “female” were reported)?*

**Answer #R4_17: We did not create a violin plot over “sex,” because a violin plot over a binary variable does not make sense.**

*7. PLOS authors have the option to publish the peer review history of their article (*[*what does this mean?*](https://journals.plos.org/plosone/s/editorial-and-peer-review-process#_blank)*). If published, this will include your full peer review and any attached files.

If you choose “no”, your identity will remain anonymous but your review may still be made public.*

***Do you want your identity to be public for this peer review?*** *For information about this choice, including consent withdrawal, please see our* [*Privacy Policy*](https://www.plos.org/privacy-policy)*.*

*Reviewer #3: No*

*Reviewer #4: No*
